# Realizing flexible bioelectronic medicines for accessing the peripheral nerves – technology considerations

**DOI:** 10.1186/s42234-018-0010-y

**Published:** 2018-06-26

**Authors:** Vasiliki Giagka, Wouter A. Serdijn

**Affiliations:** 1 Section Bioelectronics, Department of Electrical Engineering, Mathematics and Computer Science, Delft University of Technology, Delft, The Netherlands; 2 Technologies for Bioelectronics Group, Department of System Integration and Interconnection Technologies, Fraunhofer Institute for Reliability and Microintegration IZM, Berlin, Germany

## Abstract

Patients suffering from conditions such as paralysis, diabetes or rheumatoid arthritis could in the future be treated in a personalised manner using bioelectronic medicines (BEms) (Nat Rev Drug Discov 13:399–400, 2013, Proc Natl Acad Sci USA 113:8284–9, 2016, J Intern Med 282:37–45, 2017). To deliver this personalised therapy based on electricity, BEms need to target various sites in the human body and operate in a closed-loop manner. The specific conditions and anatomy of the targeted sites pose unique challenges in the development of BEms. With a focus on BEms based on flexible substrates for accessing small peripheral nerves, this paper discusses several system-level technology considerations related to the development of such devices. The focus is mainly on miniaturisation and long-term operation. We present an overview of common substrate and electrode materials, related processing methods, and discuss assembly, miniaturisation and long-term stability issues.

## Background

**Fig. 1:**
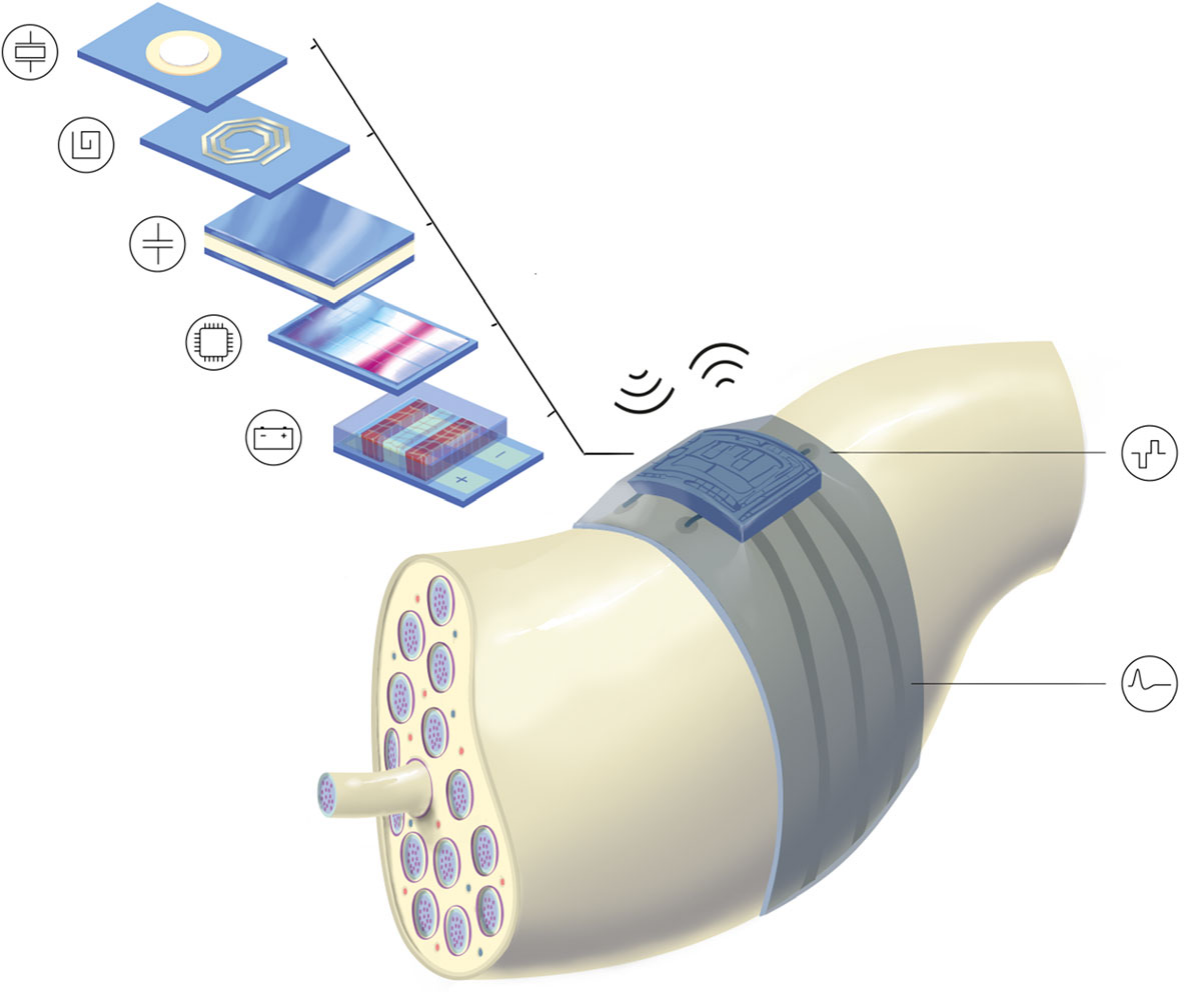
Artistic impression of a flexible BEm for accessing the peripheral nerves (concept). The BEm features a compact layout, with all components required for its wireless closed-loop operation integrated on the device. Small and thin ASICs connect to electrodes and give the system its intelligence. They receive and store energy, communicate with the outside world, process the acquired signals, and deliver the personalised therapy in the form of electrical stimuli

In the context of this paper, bioelectronic medicines are devices that use electricity to regulate biological processes, treat diseases, or restore lost functionality (Birmingham et al. 2013; Koopman et al. 2016; Bouton 2017). BEms can interact with excitable tissue in three distinct manners: they can induce, block and sense electrical activity. Depending on the application, i.e., the specific disease being treated, a combination of these functionalities might be necessary. For example, a system offering complete control over the urinary tract could require initiation of voiding (activation) in a timely manner using feedback (sensing), while avoiding involuntary voiding at all other times (blocking). However, the human urinary tract is complex and sophisticated; it includes our kidneys, ureters, bladder and urethra, and its natural control mechanisms pass through both the central as well as the peripheral nervous system (PNS). Restoration of full control of the urinary tract could therefore be possible via a variety of approaches; some of those could require a distributed wireless system of BEms, in which each unit is responsible for a specific functionality, but all are communicating and working together in a coordinated fashion to deliver the required therapy. For such an implementation, miniature (<1cm^3^) BEms placed close to their targeted nerves are required. These BEms, belong to a new generation of implants: they have to be of a compact unibody design (including a three-dimensional neural interface and the electronics), operate wirelessly (either standalone or in a distributed system), and in a closed-loop fashion, having stimulating, biomarker sensing and neural recording possibilities. Figure 1 illustrates an artistic impression of such a system.

### Interfacing the peripheral nervous system

The PNS consists of neurons whose bodies lie in the spinal cord but axons extend all the way to target organs. These afferent (sensory) or efferent (motor) nerve fibres can be myelinated or unmyelinated and are grouped together into fascicles, based on their destination rather than their function. A collection of several fascicles makes up a nerve. The organisation of fibres into fascicles changes along the length of a nerve, from fewer, larger and fused fascicles proximally, to a higher number of smaller fascicles distally. Also, the overall size and composition of peripheral nerves varies greatly; for example, the major and minor diameters of the compound femoral nerve are about 10.5 mm and 2.3 mm, respectively. In contrast, the diameter of pelvic splanchnic nerve bundles can be smaller than 0.05 mm, or larger than 0.2 mm (Gustafson et al. 2009; Jang et al. 2015).

**Fig. 2:**
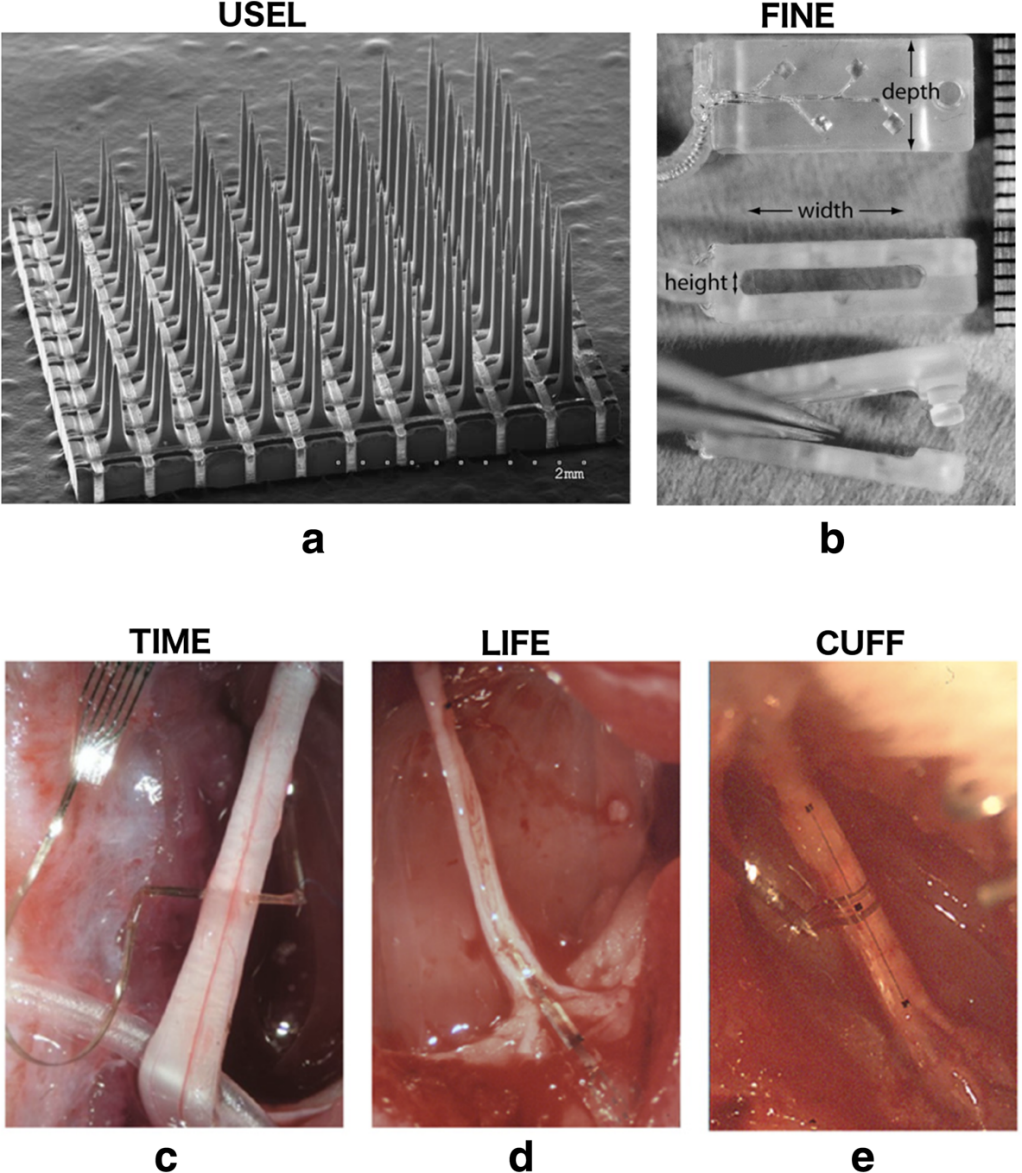
Examples of various interfaces that have been used to access the peripheral nerves. **a** A silicon-based penetrating electrode array, the Utah Slanted Electrode Array, reproduced from (Branner et al. 2004), **b**–**e** polymer-based FINE, TIME, LIFE, and CUFF electrode arrays, reproduced from (Schiefer et al. 2010) and (Badia et al. 2011). The scale bar for **b** is on the right of the picture and is in mm. **c**–**e** are all implanted in the rat sciatic nerve. **a**–**e** have been reproduced with permission

Access to the PNS can be achieved at a varying degree of invasiveness and a varying degree of selectivity. Extraneural interfaces leave the nerve intact but usually target several bundles of axons or fascicles within it, hence offer lower selectivity. Such interfaces can be placed around or through a nerve root (book electrodes), on a nerve (epineural) or around it (cuff, helicoidal, FINE^1^) (Brindley 1972; Koller et al. 1992; Loeb and Peck 1996; Tyler and Durand 2002). When higher selectivity is required, interfaces tend to be more invasive. Approaches include minor penetration of the nerve without penetrating the fascicles (SPINE^2^); longitudinal (LIFE^3^) or transverse (TIME^4^) penetration of fascicles (Tyler and Durand 1997; Lawrence et al. 2003; Boretius et al. 2010; Badia et al. 2011), to access fibres of a single or multiple fascicles, respectively; as well as single units or arrays of needle-like interfaces that penetrate fascicles and aim to access even single fibres (Utah, Michigan and Twente arrays) (Branner et al. 2001; Anderson et al. 1989; Rutten and Meier 1991). Figure 2 illustrates some examples of interfaces that have been used to access the PNS. For a thorough review on the subject the reader is referred to (Navarro et al. 2005).

### Stiff versus flexible materials

The hardware implementation of BEms, including the design, material selection and fabrication process, is largely dictated by the implantation site. More specifically, the size of the targeted tissue, its location within the body (e.g., deep vs close to the skin), the available area and anatomical structures around it (e.g., soft tissue, bones), the related mechanics, perfusion rates, as well as the surgical procedures, will all place additional requirements that the BEm needs to adhere to. BEms can be constructed from hard or softer materials, which define the implant’s mechanical properties.

Hard implants have been extensively used for probes penetrating the brain (Moxon et al. 2004; Vetter et al. 2004; Rousche and Normann 1997), while they are also very well suited for attachment on hard structures inside the body, such as on teeth or bones, due to their similar mechanical properties, reflected by their Young’s moduli. Silicon-based implants have the advantage of a fabrication process that is often compatible with standard complementary metal–oxide–semiconductor (CMOS) processes, resulting in easier electronic integration, with high yield and a high degree of miniaturisation. They are, however, stiff and rigid, and micromotion of the device might cause tissue damage (Alexander Arts et al. 2003). Despite the fact that modified designs tailored to the peripheral nerves have shown promising performance in long-term tests (Branner et al. 2004; Christenses et al. 2016), their use in the PNS is limited.

Softer materials match much better the mechanical properties of the surrounding tissue, and several of them have shown excellent performance during chronic implantation, with only mild to minor foreign body reactions (25). These properties are particularly important when designing implants for accessing the PNS. Interfacing with the peripheral nerves is challenging due to their often small size and fragile nature. State-of-the-art implants that target the PNS are, therefore, mainly based on flexible materials (Navarro et al. 2005). Many of them have shown promising performance in long-term in vivo tests (Sohal et al. 2016; Caravaca et al. 2017; Hara et al. 2016; Wurth et al. 2017; Pothhof et al. 2016). The majority of those implants are however passive devices, connected to remote electronics using long wires.

### BEms are active implants

The envisioned operation of BEms is to deliver personalised therapy, which will require adjustment of the treatment and dosage, in a similar fashion as with conventional drugs (Birmingham et al. 2014). To this end, bi-directional communication and programmability are necessary, while safe and secure chronic operation is of utmost importance. Despite the fact that some on-site intelligence can be incorporated with the use of flexible transistors (Viventi et al. 2011), the level of maturity of such technologies is not yet sufficient to meet neither the foreseen processing complexity nor the strict requirements for clinical use. For these, high performance active electronics are necessary. Systems assembled from off-the-shelf components could provide a rapid prototyping solution, but these are neither area nor power optimised. We thus envision that application specific integrated circuits (ASICs) will be an integral part of the implant.

The development of active implants is, in many ways, more challenging compared to their passive counterparts. Even though integrated circuit fabrication technologies are well developed and characterised, hence high performance ASICs can be reliably made at high yields, translating these into implantable microsystems presents its own additional challenges, which, at the moment, do not have a universal solution. Referring to our envisioned flexible BEm for the PNS, illustrated in Fig. 1, and targeting a decades-long lifetime, we identify the following challenges: (a) ensuring that the presence of the implant does not cause any adverse effects to the tissue; (b) ensuring that the implant does not cause any adverse effects to the tissue during active operation; (c) achieving the spatial selectivity required for the application; (d) preserving the flexibility (or bendability) of the implant despite the rigid silicon ASIC; (e) reliably connecting the ASIC to the electrodes so that failure due to movement does not occur; (f) reliably protecting the ASIC and interconnects from the body so that failure does not occur; and (g) bringing enough energy deep inside the body to reach the BEm, while still keeping the volume of the implant small. Several of the above are shared challenges between passive and active implants, while some others are mainly application dependent. In this paper, we focus on the relevant technology considerations associated with the hybrid nature of these devices. We begin our discussion with common materials used as substrates and their processing and electrode requirements, before presenting the challenges related to the assembly, miniaturisation, and long-term stability of these hybrid implants.

## Technology considerations

### Polymers as substrates

Existing technologies for realising flexible implants are mainly polymer-based. These polymers are often used as substrates, but also as intermediate insulation or passivation layers. Most commonly used polymers are polyimide (Rousche et al. 2001; Rodriguez and al 2000; Lacour et al. 2008; Cutrone et al. 2015), parylene (Takeuchi et al. 2005; Kuo et al. 2013; Sohal et al. 2014), polydimethylsiloxane (PDMS) (Minev et al. 2015; Schuettler et al. 2005a; Guo et al. 2013), but several others have been reported, such as liquid crystal polymer (LCP) (Wang et al. 2009; Gwon et al. 2016), SU-8 photoresist (Altuna et al. 2012; Huang et al. 2014), polyurethane (Roger et al. 2016). These polymers come in different forms, including powder (parylenes), liquid (polyimides, PDMS, SU-8), and sheet or film (polyimides, LCPs, polyurethanes, SU-8). Depending on the material, common deposition techniques include spin-coating, vapour deposition and melting. Polymeric substrates can be patterned using laser ablation (PDMS, LCP), wet or dry etching (PDMS), reactive ion etching (polyimides, parylene, LCP). Several of these materials come in photopatternable versions (PDMS, polyimide, SU-8), although none of those is approved for use in chronic implants at the moment. Resulting layer thicknesses range from hundreds of nanometres (parylene), via some or tens of micrometres (polyimide, parylene, PDMS, SU-8, LCPs), up to some millimetres (LCPs). For a more detailed and complete discussion on polymer processing for implants the reader is referred to (Hassler et al. 2010; Scholten and Meng 2015).

Challenges of the use of polymers as substrate materials for BEms relate to their limited working temperatures (< 350 °C), which makes them incompatible with several important processing methods, such as chemical vapour deposition and baking, but also with conventional assembly methods, such as bonding and soldering. Further processing challenges relate to the usually slow polymer etching. Processing practices can sometimes adversely affect the biocompatibility as well as the stability of the implant in the body (Schuettler et al. 2005b).

Polymers are permeable to gasses and water vapour, which often means that additional protection of structures and components is necessary to ensure decade-long operation inside the body – more on this is discussed later in the paper. Air can be trapped in the polymer during fabrication and manifest itself as air bubbles, which can gradually compromise adhesion between layers and promote delamination.

Furthermore, the mechanical properties of these flexible materials, although they match those of human tissue better, complicate handling and insertion into the body. Therefore, additional design features and custom-made tools, such as stylets, may have to be employed to ease implantation.

### Electrodes

Electrodes are necessary interfaces in all medical devices that act on the electrophysiology of the body, with an, often, dual functionality: they are used to inject electrical signals into the human tissue, but also to sense electrical activity from the tissue. Generally, denser electrode integration is desirable as it offers higher spatial resolution, both for the stimulating and sensing operation mode. Noble metals such as gold and platinum have been extensively used as electrode materials.

#### Size

One major bottleneck limiting the achievable electrode density is that small electrode sizes come at the expense of higher impedances, an undesired feature both in the recording as well as in the stimulation domain.

In the recording domain, the higher electrode impedances will result in a lower signal-to-noise ratio, making it harder to extract the low amplitude extracellular signals from the noisier environment.

In the stimulation domain, a certain amount of electrical charge is required to activate excitable tissue. High electrode impedances would result in large voltages appearing across the electrode-tissue interface, which could trigger undesirable electrochemical reactions (e.g. electrolysis). These electrochemical reactions might be harmful to the tissue or damage the electrode. Furthermore, large electrode-tissue voltages complicate the design of the output-stage electronics, as they necessitate the use of high-voltage output transistors, which are large, and often incompatible with the smaller CMOS technology nodes used for fast digital circuits. Solutions to this include implementing the whole design in larger technologies (Giagka et al. 2015a; Liu et al. 2012) at the expense of slower speed and increased power consumption, or splitting it into a two-chip solution (Ethier and Sawan 2011), requiring larger total area and a more complicated assembly.

Some techniques exist that aim to reduce the electrode impedance without increasing their size. One popular technique is roughening the surface of electrodes, in order to increase the electrochemical surface area (Cogan 2008), while keeping the geometric surface area constant. Alternatively, electrodes are often coated with more suitable materials, which, depending on the application, aim to either increase the charge injection capacity or improve the noise performance. Possible candidates include metals, conducting polymers, and more recently carbon, as well as combinations of the above. More specifically, platinum black (Tand et al. 2014) and nanostructured platinum grass (Liu et al. 2012) coatings have both shown significant improvements in electrode impedance, due to an increased surface area. The deposition processes for platinum black have reportedly caused cytotoxic reactions due to lead traces found in the electroplating electrolyte (Schuettler et al. 2005b). This was not observed for nanostructured platinum grass (Boehler et al. 2015). Iridium oxide (IrO_2_) coating is corrosion resistant and exhibits, in general, good biocompatibility and higher charge delivery capacity (Wang et al. 2009). Nevertheless, its exact properties depend greatly on the fabrication method – activated, sputtered and electrodeposited variants have been reported – which, in turn, affects the stability of the coating. Titanium nitride (TiN) coated electrodes have exhibited good electrochemical stability and charge injection limits similar to that of iridium oxide, but the biocompatibility of the coating still needs to be investigated further (Brunton et al. 2015). Poly (3,4 – ethylene dioxytiophene) (PEDOT) is a conductive polymer known to exhibit low electrochemical impedance due to its porous film surface. PEDOT is interesting as it can be modified by the addition of bioactive molecules to e.g. promote cell adherence to the electrode surface. It exhibits large charge injection capacity and has proven to be stable in vivo without causing any toxic effect (Charkhar et al. 2015). More recently, looking for ways to improve the stability of the neural interface by creating a friendly environment for cells, carbon-based materials, such as carbon nanotubes and graphene also have been investigated for neural applications (Yi et al. 2015; Lu et al. 2016). Despite the fact that there is no consensus yet regarding their biocompatibility, both materials have shown promising performance. Carbon nanotubes are interesting due to their high surface-to-volume ratio, while graphene’s attractiveness is due to its combined properties of transparency, conductivity and mechanical strength. Combinations of the above materials have also been reported for electrode coating. For a more thorough review on the topic the reader is referred to (Chen et al. 2017).

#### Number of electrodes

Another factor limiting the electrode density is the number of connections required to access each of them independently.

Although systems with thousands of electrodes have been reported (Dragas et al. 2017), these are silicon-integrated systems meant for in vitro recordings, with electrodes and electronics monolithically integrated. Current state-of-the-art silicon-based in vivo systems do not commonly feature more than hundreds of electrodes (Campbell et al. 1991), with recent improvements bringing this number closer to one thousand (Shobe et al. 2015; Scholvin et al. 2016; Buszáki et al. 2015), even on a single probe (Jun et al. 2017). For systems on flexible substrates this number is usually in the order of tens for similar areas (Rubehn et al. 2009). Denser electrode integration can be achieved using multiple metal layers (Suaning et al. 2007), but flexible systems are rarely multi-layered, due to the more complex fabrication process this implies and the higher chance of failure. In another approach, the use of flexible silicon electronics has been successfully employed to map brain activity in vivo over larger areas (Viventi et al. 2011), but stimulation and long-term performance are yet to be demonstrated.

Based on the above, it is foreseen that the first generation of flexible BEms will feature several, up to tens of electrodes, rather than hundreds, with relatively larger sizes, recording compound neural activity as closed-loop control signals, rather than single-unit action potentials – which are anyway difficult to acquire chronically.

### Interconnects

In virtually all neural implants reported to date there is a strong need for long interconnect lines (connecting electrode sites with the driving or read-out electronics). Materials that have been commonly used for such purposes on flexible substrates are noble metals and alloys, such as platinum, platinum-iridium, gold.

The density of these interconnect lines largely depends on the selected technology and processing, but reported examples include: laser-patterned platinum, or platinum iridium foil tracks (usually 12 μm thin) on PDMS (Schuettler et al. 2005a), lithographically-patterned gold or platinum thin (300 nm) films on polyimide or parylene (Rodriguez and al 2000), thermally evaporated ultra-thin (35 nm) stretchable gold tracks on PDMS (Minev et al. 2015).

Generally, although platinum is preferred as stimulating electrode material due to its higher charge storage capacity, its low conductivity (more than four times lower compared to gold) renders it undesirable for narrow, thin and long interconnects. Platinum is therefore, often, combined with gold to circumvent this problem. It is generally desired that dissimilar metals are avoided, as galvanic corrosion could occur if exposed to body liquid. However, as the galvanic potentials of gold and platinum are close to each other, such failures for this intermetallic combination are unlikely to occur.

Due to the softer nature of the polymeric substrates, in which these interconnects need to be embedded, however, deformation due to motion from nearby organs is likely to occur. The degree of this deformation will of course vary depending on the implantation site. Therefore, the mechanical properties of these interconnects (and their substrates) should be such that they allow the implant to withstand the expected deformation over millions or even billions of cycles without failing.

These mechanical properties depend on the initial form of the material as well as its processing techniques. In the foil form, platinum is often mixed with iridium for reinforcement. This solid alloy is usually used in thicknesses in the order of 10 μm (Giagka et al. 2015b), which offers a good compromise between mechanical stability and ease of processing. When nm-thin layers of metals are used as interconnects, these are usually constructed by sputtering, or evaporation of metal particles in an additive process. The resulting layer’s cohesive strength (and conductivity) is worse than that of an equal-thickness layer of solid material (Ordonez et al. 2012). Nevertheless, evaporated thin-film gold layers on PDMS have shown to remain electrically conductive when stretched by tens of percent, due to bridging of built-in microcracks (Lacour et al. 2006).

Due to the presence of body fluids, adhesion between metal layers and the polymeric substrates can be compromised in the long-term. When metal foils are used, a possible way to improve long-term stability is to perforate and mechanically lock them in place by the top and bottom polymer layers, thus avoiding metal dislocation (Ordonez et al. 2012).

Nevertheless, when current is allowed to pass through these tracks into the ionic body solution, at high current densities, metal corrosion will slowly occur. Loss of adhesion has much more prominent effects when nanometer films are used, as complete dissolution of the thin metal and implant failure is expected. It is therefore not surprising that researchers have focused on polymer-to-metal adhesion promotion techniques. These include oxygen plasma treatment (Rubehn et al. 2009), extra titanium or chromium metallization layers, as well as silicon carbide additional insulating coating (Cogan et al. 2003).

### Thin electronics

Common thicknesses of polymeric substrates are usually in the range of tens of micrometres. Although thicker substrates can be fabricated, thinner devices are preferred as these usually can withstand larger bending radii. The hybrid nature of flexible BEms implies that the custom designed ASICs required for the performance of the system will have to be assembled on the flexible substrates in a heterogeneous process.

Typical thicknesses of fabricated chips are in the order of 500 μm – 1 mm, while the active layers may occupy only the top 5–10 μm, depending on the complexity of the design. The extra material, which mainly serves as mechanical support, can be removed after processing to create thinner, hence bendable, devices. Chips with functional structures thinner than 25 μm have been reported (Burghartz et al. 2010). Several techniques for silicon back-thinning have been developed from a combination of different mechanical and chemical processes. Most commonly, grinding, lapping or polishing are used for the removal of the major part of the material, usually followed by a stress-relief step. In this step dry, wet or laser chemical etching techniques are employed for the removal of the last 10–100 μm of silicon, to reduce the backside damage caused by the previous coarse step and ensure a smooth surface finish (Feil et al. 2003). In nearly all the efforts described above, the problem of silicon thinning is dealt with at wafer level. Die level thinning has also been reported for prototyping purposes, when ASICs come from multi-project wafers (Giagka et al. 2014).

However, even after thinning and die separation, millimeter-small, micrometer-thin individual dice have to be handled, mounted, aligned and assembled on the flexible substrates, and related challenges are far from trivial as they are radically different from wafer level processes (Bosman et al. 2011).

Thinned ASICs will add to the flexibility of the BEm, but may have to be bent and conform to the features or structures of the implantation site. Care should therefore be taken that the resulting mechanical strength of the thin die is adequate for the application. This has been greatly correlated with the surface topology of an ASIC, with smoother surfaces presenting greater strength (Kröninger and Mariani 2006).

Bending will also affect the performance of the ASIC. Several groups have started looking into this, and new challenges lie ahead in the integrated circuit design domain for implantable ASICs; expected bending should be characterized, transistor models and design kits should be adapted to include expected performance changes (Vilouras et al. 2017), and new, robust circuits should be designed such that their performance will remain immune to bending.

### Limited off-chip components

Due to the limited available space around the peripheral nerves it is desirable that the majority of components required for the operation of the system are integrated on chip, as in Fig. 1. Contrary to existing neuromodulation implants, conventional large blocking capacitors will be close to impossible to fit inside the BEm. Complete elimination of blocking capacitors could be an option, as they anyway do not offer the protection the community thought they did–they introduce a voltage offset across the electrode-tissue interface, which is often neglected and needs to be properly characterised to ensure safe operation (van Dongen and Serdijn 2016). Blocking capacitors are still useful to block direct currents (DCs), but fail-safe blocking capacitor-free stimulator designs have been reported (Liu et al. 2008; Sit and Sarpeshkar 2007), and could be employed instead. When large capacitors are still needed, for example for energy storage purposes, silicon integration of such structures could be another viable alternative (Lallemand and Voiron 2013). Other common off-chip components include light emitting diodes (LEDs) for optogenetic stimulation, and receiving elements for wireless power transfer, such as inductors, antennas, ultrasonic transducers. Efforts have been reported to integrate all of these on chip for mm-scale devices (Zargham and Glenn Gulak 2015; Gurun et al. 2014).

### ASIC assembly

When electronics are integrated on implantable devices the connections need to provide both good electrical and mechanical stability to prevent implant failure throughout its lifetime. The most common assembly techniques used in the microelectronics industry are wire bonding, tape automated bonding and flip-chip bonding. In a hybrid system, as the one discussed here, the thin silicon chip assembly needs to be performed on a flexible substrate. The two materials will have very different mechanical properties and high temperatures like the ones used for thermocompression bonding are often above the substrate’s melting point. Most of the aforementioned processes have been adjusted for use on flexible substrates while at the same time some other processes have been developed.

Gold-wire bonding onto flexible polymeric substrates (polyimides, thermoplastics and polytetrafluoroethylene (PTFE)) has been reported (Hall et al. 1996; Karnezos et al. 1996).

Flip-chip bonding has been extensively used on flexible substrates in several variations, including reflow soldering and thermocompression processes for thin dice (50 μm) (Banda et al. 2008; Zhang et al. 2009), but dispensing the underfill without allowing the material to flow on top of the very thin die is challenging. Polymeric adhesives are also very popular for flip-chip bonding on flexible substrates (Lai and Liu 1999; Chen et al. 2000; Lu and Chen 2010; Chiang et al. 2006; Chuang et al. 2010) as they can eliminate the need for underfill. The adhesive acts as the interconnection medium between the bumped chip and the substrate, while it at the same time protects the contacts and provides mechanical support. Transfusion flip-chip bonding has also been reported on flexible substrates (Kulojarvi and Kivilahti 1998).

Gold electroplating (Govaerts et al. 2009) and conductive pastes in micromachined trenches (Marinov et al. 2012) have also been employed to electrically integrate thinned chips into polyimide substrates.

When thick film rather than thin film is used options could include soldering, which provides reliable results, although it would probably not be suitable for very fine pitches (Schuettler et al. 2008), parallel-gap welding (Schuettler et al. 2008) and laser welding, which can be used to weld dissimilar materials with very different thicknesses (ratio 50:1) (Rischall and Shackleton 1964).

Another interesting approach is the microflex assembly technique (Stieglitz et al. 2000), which is based on biocompatible materials and can be used both with thick but also thin films. In this process, also referred to as electrical rivet bonding, conductive tracks are thermosonically bonded on a substrate using gold ball studs through via holes, as microrivets.

### Protection of electronics

BEms are developed to live decades in the human body and it is essential that they are reliably protected from the surrounding environment.

In-body protection of implantable electronics has mostly been realised using hermetically sealed metallic or ceramic enclosures, with electrical feedthroughs for connections to electrodes and other remote parts.

This protection mechanism aims to prevent corrosion by keeping the internal environment of the enclosure dry. The hermeticity of such packages can be characterised, and predictions regarding the expected device lifetime can be extracted.

However, for the small dimensions of BEms, conventional protection is becoming increasingly difficult to implement. Furthermore, rigid packages are likely to damage the fragile targeted nerves, while it has been shown that for hermetic packages with volumes less than 1mm^3^, long-life predictability is lost (Vanhoestenberghe and Donaldson 2013).

It is, therefore, natural that several groups have been investigating other options, based on encapsulation. In encapsulation, the encapsulant attaches to the surface of all components to be protected, forming a conformal layer which protects them from the formation of liquid water, thereby preventing corrosion. Different materials can be used as encapsulants. Candidates include polymers, despite the fact that they are permeable to water vapour. As an example, PDMS-coated sacral nerve roots stimulators made by Finetech Medical, UK, have lived in the human body for decades (Rijkhoff 2004).

Polymer encapsulation relies on achieving good adhesion between the substrate and encapsulant, which, if not compromised, should provide reliable protection. Therefore, clean substrates and void-free polymers are crucial. Furthermore, implantable ASICs, should be designed such that the current density through their passivation layer is limited, as this has been shown to affect the lifetime (Vanhoestenberghe and Donaldson 2013).

Another approach that is currently being explored is a combination of the above, in which ceramic atomic layer deposition (ALD) layers, acting as barriers, are sandwiched between layers of polymer, to increase softness and flexibility (Op de Beeck et al. 2017; Xie et al. 2014). This approach employs redundancy in an attempt to delay failure and to ensure long-term reliability of the protection in vivo, and has shown promising results in soak tests. Eventually, deformation, bending, and application of DC biases need to also be incorporated in soak tests to fully characterise the long-term performance of these protection methods.

## Conclusion

In this paper we discussed some of the most prominent technological considerations related to the development of flexible BEms for accessing the peripheral nervous system. This review is not meant to be exhaustive, but rather to give the reader a good round image of the related system-level technological challenges. We have focused on challenges related to the hybrid nature of these active microsystems, when miniaturization and reliable long-term operation are the goals. Technologies for developing flexible implants are clearly focusing on polymer processing, with some clear prominent candidates standing out because of their proven biocompatibility. Nevertheless, substrate processing steps need careful consideration, as they might adversely affect both the reliability, but also the overall biocompatibility of the implant. Electrode materials have moved from noble metals to advanced coatings, constructed by either different materials, or different geometries. Several of those exhibit good performance in vivo, both in the recording as well as in the stimulation domain. Their reduced impedance and/or increased electrochemical surface allow for denser electrode integration, hence higher specificity and further miniaturisation. For the targeted decade-long lifetime though, further characterization and testing is often necessary. Interconnecting lines are always an unwanted but necessary feature in all implantable devices. In flexible BEms, these need to be strong but at the same time well protected to avoid failures. Similarly, new protection methods for any active electronics are necessary. Large metallic or ceramic hermetic packages do not any longer fit in the miniature dimensions discussed here. Promising material candidates that could offer this protection include combinations of polymers and ALD ceramics.

In summary, this review discussed developments related to the need for miniaturization and safe long-term operation. Challenges, however, do not stop here. Many other major aspects, which were out of the scope of this review, remain unsolved. These include: (a) how to bring enough power inside the body to operate such systems wirelessly, (b) how to map the functions of body organs or body processes onto specific neurons or nerves, (c) how to use biosensing to regulate the therapy, (d) how to guarantee the safety and security of the users. New results and state-of-the-art works that try to tackle several of these aspects are continuously being reported. Overall, combined efforts from different fields are necessary to make these systems a reality.

## Footnotes

1 Flat interface nerve electrode

2 Slowly penetrating interfascicular nerve electrode

3 Longitudinal intrafascicular electrode

4 Transverse intrafascicular multichannel electrode

## Authors’ information

VG is Assistant Professor of Bioelectronics at Delft University of Technology, Delft, The Netherlands, and she is also leading the group Technologies for Bioelectronics, at Fraunhofer Institute for Reliability and Microintegration IZM, Berlin, Germany. Between her two affiliations she is carrying out research on the design and fabrication of active implantable microsystems. WS is full Professor at Delft University of Technology, Delft, The Netherlands where he heads the Section Bioelectronics. His research interests are on integrated biomedical circuits and systems. He is an IEEE Fellow, an IEEE Distinguished Lecturer and a mentor of the IEEE.

## References

[CR1] Alexander Arts H, Jones DA, Anderson DJ (2003). Prosthetic stimulation of the auditory system with intraneural electrodes. Ann Otol Rhinol Laryngol.

[CR2] Altuna A (2012). SU-8 based microprobes with integrated planar electrodes for enhanced neural depth recording. Biosens Bioelectron.

[CR3] Anderson DJ (1989). Batch-fabricated thin-film electrodes for stimulation of the central auditory system. IEEE Trans Biomed Eng.

[CR4] Badia J (2011). Comparative analysis of transverse intrafascicular multichannel, longitudinal intrafascicular and multipolar cuff electrodes for the selective stimulation of nerve fascicles. J Neural Eng.

[CR5] Banda C, Johnson RW, Zhang T, Hou Z, Charles HK (2008). Flip chip assembly of thinned silicon die on flex substrates. IEEE Trans Electron Pa M.

[CR6] Birmingham K (2013). Bioelectronic medicines: a research roadmap. Nat Rev Drug Discov.

[CR7] Birmingham K (2014). Bioelectronic medicines: a research roadmap. Nat Rev Drug Discov.

[CR8] Boehler C, Stieglitz T, Asplund M (2015). Nanostructured platinum grass enables superior impedance reduction for neural microelectrodes. Biomaterials.

[CR9] Boretius T (2010). A transverse intrafascicular multichannel electrode (TIME) to interface with the peripheral nerve. Biosens Bioelectron.

[CR10] Bosman E (2011). Ultrathin optoelectronic device packaging in flexible carriers. IEEE J Sel Top Quant.

[CR11] Bouton C (2017). Cracking the neural code, treating paralysis and the future of bioelectronic medicine. J Intern Med.

[CR12] Branner A, Stein RB, Fernandez E, Aoyagi Y, Normann RA (2004). Long-term stimulation and recording with a penetrating microelectrode array in cat sciatic nerve. IEEE Trans Biomed Eng.

[CR13] Branner A, Stein RB, Normann RA (2001). Selective stimulation of cat sciatic nerve using an array of varying-length microelectrodes. J Neurophysiol.

[CR14] Brindley GS (1972). Electrode array for making long-lasting electrical connections to spinal roots. J Physiol.

[CR15] Brunton E (2015). In vivo comparison of the charge densities required to evoke motor responses using novel annular penetrating microelectrodes. Front Neurosci.

[CR16] Burghartz J, Appel W, Harendt C, Rempp H, Richter H (2010). Ultra-thin chip technology and applications, a new paradigm in silicon technology. Solid State Electron.

[CR17] Buszáki G (2015). Tools for probing local circuits: high-density silicon probes combined with optogenetics. Neuron.

[CR18] Campbell PK, Jones KE, Huber RJ, Horch KW, Normann RA (1991). A silicon-based, three-dimensional neural inreface: manufacturing processes for an intracortical electrode array. IEEE Trans Biomed Eng.

[CR19] Caravaca AS (2017). A novel flexible cuff-like microelectrode for dual purpose, acute and chronic electrical interfacing with the mouse cervical vagus nerve. J Neural Eng.

[CR20] Charkhar H (2015). Chronic intracortical neural recordings using microelectrode arrays coated with PEDOT-TFB. Acta Biomater.

[CR21] Chen KY, Zenner RLD, Arneson M, Mountain D (2000). Ultra-thin electronic device package. IEEE Trans Adv Packag.

[CR22] Chen N (2017). Neural interfaces engineered via micro- and nanostructured coatings. Nano Today.

[CR23] Chiang WK, Chan WC, Ralph B, Holland A (2006). Processability and reliability of nonconductive adhesives (NCAs) in fine-pitch chip-on-flex applications. J Electron Mater.

[CR24] Christenses MB, Wark HAC, Hutchinson DT (2016). A histological analysis of human median and ulnar nerves following implantation of Utah slanted electrode arrays. Biomaterials.

[CR25] Chuang CL, Liao QA, Li HT, Liao SJ, Huang GS (2010). Increasing the bonding strength of chips on flex substrates using thermosonic flip-chip bonding process with nonconductive paste. Microelectron Eng.

[CR26] Cogan S, Edell DJ, Guzelian AA, Liu YP, Edell R (2003). Plasma-enhanced chemical vapour deposited silicon carbide as an implantable dielectric coating. J Biomed Mater Res A.

[CR27] Cogan SF (2008). Neural stimulation and recording electrodes. Annu Rev Biomed Eng.

[CR28] Cutrone A (2015). A three-dimensional self-opening intraneural peripheral interface (SELINE). J Neural Eng.

[CR29] Dragas J (2017). In vitro multi-functional microelectrode array featuring 59760 electrodes, 2048 electrophysiology channels, stimulation, impedance measurement, and neurotransmitter detection channels. IEEE J Solid St Circ.

[CR30] Ethier S, Sawan M (2011). Exponential current pulse generation for efficient very high-impedance multisite stimulation. IEEE Trans Biomed Circuits Syst.

[CR31] Feil M (2003). Ultra thin ICs and MEMS elements: techniques for wafer thinning, stress-free separation, assembly and interconnection. Microsyst Technol.

[CR32] Giagka V, Demosthenous A, Donaldson N (2015). Flexible active electrode arrays with ASICs that fit inside the rat’s spinal canal. Biomed Microdev.

[CR33] Giagka V, Eder C, Donaldson N, Demosthenous A (2015). An implantable versatile electrode-driving ASIC for chronic epidural stimulation in rats. IEEE Trans Biomed Circuits Syst.

[CR34] Giagka V, Saeidi N, Demosthenous A, Donaldson N. Controlled silicon IC thinning on individual die level for active implant integration using a purely mechanical process. Proc. IEEE ECTC 2014, Orlando, FL, USA, pp. 2014. 2213–2219.

[CR35] Govaerts J, Christiaens J, Bosman E, Vanfleteren J (2009). Fabrication processes for embedding thin chips in flat flexible substrates. IEEE Trans Adv Packag.

[CR36] Guo L (2013). A PDMS-based integrated stretchable microelectrode array (isMEA) for neural and muscular surface interfacing. IEEE Trans Biomed Circuits Syst.

[CR37] Gurun G (2014). Single-chip CMUT-on-CMOS front-end system for real-time volumetric IVUS and ICE imaging. IEEE Trans Ultrason Ferroelectr Freq Control.

[CR38] Gustafson KJ (2009). Fascicular anatomy of human femoral nerve: implications for neural prostheses using nerve cuff electrodes. J Rehabil Res Dev.

[CR39] Gwon TM, Kim C, Shin S, Park JH, Kim J, Kim SJ (2016). Liquid crystal polymer (LCP)-based neural prosthetic devices. Biomed Eng Lett.

[CR40] Hall E, Lyons AM, Weld JD (1996). Gold wire bonding onto flexible polymeric substrates. IEEE Trans Compon Packag Manuf Technol A.

[CR41] Hara SA (2016). Long-term stability of intracortical recordings using perforated and arrayed Parylene sheath electrodes. J Neural Eng.

[CR42] Hassler C, Boretius T, Stieglitz T (2010). Polymers for neural implants. J Polym Sci Part B Polym Phys.

[CR43] Huang AH, Lin AP, Chen JJJ (2014). In vitro and in vivo characterization of SU-8 flexible neuroprobe: from mechanical properties to electrophysiological recording. Sensors Actuators A.

[CR44] Jang HS (2015). Composite nerve fibers in the hypogastric and pelvic splanchnic nerves: an immunohistochemical study using elderly cadavers. Anatomy Cell Biology.

[CR45] Jun JJ (2017). Fully integrated silicon probes for high-density recording of neural activity. Nature.

[CR46] Karnezos M, Goetz M, Dong F, Ciaschi A, Chidambaram N. Flex tape ball grid array. Proc. IEEE ECTC 1996, Orlando, FL. 1996. pp. 1271–7.

[CR47] Koller R (1992). Long-term results of nervous tissue alterations caused by epineurial electrode application: an experimental study in rat sciatic nerve. Pacing Clin Electrophysiol.

[CR48] Koopman F (2016). Vagus nerve stimulation inhibits cytokine production and attenuates disease severity in rheumatoid arthritis. Proc Natl Acad Sci U S A.

[CR49] Kröninger W, Mariani F. Thinning and singulation of silicon: root causes of the damage in thin chips. Proc. IEEE ECTC 2006, San Diego, CA, USA. 2006. pp. 1317–22.

[CR50] Kulojarvi K, Kivilahti K (1998). A low temperature interconnection method for electronics assembly. IEEE Trans Compon Packag Manuf Technol A.

[CR51] Kuo JTW (2013). Novel flexible Parylene neural probe with 3D sheath structure for enhancing tissue integration. Lab Chip.

[CR52] Lacour SP, Atta R, FitzGerald JJ, Blamire M, Tarte E, Fawcett J (2008). Polyimide micro-channel arrays for peripheral nerve regenerative implants. Sensors Actuators A.

[CR53] Lacour SP, Chan D, Wagner S, Li T, Suo Z (2006). Mechanisms of reversible stretchability of thin metal films on elastomeric substrates. Appl Phys Lett.

[CR54] Lai Z, Liu J (1999). Anisotropically conductive adhesive flip-chip bonding on rigid and flexible printed circuit substrates. IEEE Trans Compon Packag Manuf Technol B.

[CR55] Lallemand F, Voiron F. Silicon interposers with integrated passive devices, an excellent alternative to discrete components in Proc. Europ. Microelectr. Packag. Conf. (EMPC) 2013, Grenoble, France. 2013.

[CR56] Lawrence SM, Dhillon GS, Horch KW (2003). Fabrication and characteristics of an implantable, polymer-based, intrafascicular electrode. J Neurosci Methods.

[CR57] Liu X, Demosthenous A, Donaldson N (2008). An integrated implantable stimulator that is fail-safe without off-chip blocking-capacitors. IEEE Trans Biomed Circuits Syst.

[CR58] Liu X, Demosthenous A, Vanhoestenberghe A, Jiang D, Donaldson N (2012). Active books: the design of an implantable stimulator that minimizes cable count using integrated circuits very close to electrodes. IEEE Trans. Biomed Circuits Syst.

[CR59] Loeb GE, Peck RA (1996). Cuff electrodes for chronic stimulation and recording of peripheral nerve. J Neurosci Methods.

[CR60] Lu ST, Chen WH (2010). Reliability and flexibility of ultra-thin chip-on-flex (UTCOF) interconnects with anisotropic conductive adhesive (ACA) joints. IEEE Trans Adv Packag.

[CR61] Lu Y, Lyu H, Richardson AG, Lucas TH, Kuzum D (2016). Flexible neural electrode array based-on porous graphene for cortical microstimulation and sensing. Sci Rep.

[CR62] Marinov V (2012). Laser-enabled advanced packaging of ultrathin bare dice in flexible substrates. IEEE Trans Compon Packag Manuf Technol.

[CR63] Minev IR (2015). Electronic dura mater for long-term multimodal neural interfaces. Science.

[CR64] Moxon KA, Leiser SC, Gerhardt GA, Barbee KA, Chapin JK (2004). Ceramic-based multisite electrode arrays for chronic single-neuron recording. IEEE Trans Biomed Eng.

[CR65] Navarro X (2005). A critical review of interfaces with the peripheral nervous system for the control of neuroprostheses and hybrid bionic systems. J Peripher Nerv Syst.

[CR66] Op de Beeck M, et al.. Ultra-thin biocompatible implantable chip for bidirectional communication with peripheral nerves. In Proc. IEEE BioCAS, Turin, Italy. 2017. pp. 576–79.

[CR67] Ordonez J, Schuettler M, Boehler C, Boretius T, Stieglitz T (2012). Thin films and microelectrode arrays for neuroprosthetics. MRS Bull.

[CR68] Pothhof F (2016). Chronic neural probe for simultaneous recording of single-unit, multi-unit, and local field potential activity from multiple brain sites. J Neural Eng.

[CR69] Rijkhoff NJM (2004). Neuroprostheses to treat neurogenic bladder dysfunction: current status and future perspectives. Childs Nerv Syst.

[CR70] Rischall H, Shackleton J (1964). Laser welding for microelectronic interconnections. IEEE Trans Compon Parts.

[CR71] Rodriguez FJ, al e (2000). Polyimide cuff electrodes for peripheral nerve stimulation. J Neurosci Methods.

[CR72] Roger Y (2016). Grid-like surface structures in thermoplastic polyurethane induce anti-inflammatory and anti-fibrotic processes in bone marrow-derived mesenchymal stem cells. Colloids Surf B Biointerfaces.

[CR73] Rousche PJ, Normann RA (1997). Chronic recording capability of the Utah Intracortical electrode Array in cat sensory cortex. J Neurosci Methods.

[CR74] Rousche PJ, Pellinen DS, Pivin DP, Williams JC, Vetter RJ (2001). Flexible polyimide-based intracortical electrode arrays with bioactive capability. IEEE Trans Biomed Eng.

[CR75] Rubehn B, Bosman C, Oostenveld R, Fries P, Stieglitz T (2009). A MEMS-based flexible multichannel ECOG-electrode array. J Neural Eng.

[CR76] Rutten WL, Meier HJ (1991). Selectivity of intraneural prosthetic interfaces for muscular control. Med Biol Eng Comput.

[CR77] Schiefer MA, Polasek KH, Triolo RJ, Pinault GC, Tyler DJ (2010). Selective stimulation of the human femoral nerve with a flat interface nerve electrode. J Neural Eng.

[CR78] Scholten K, Meng E (2015). Materials for microfabricated implantable devices. Lab Chip.

[CR79] Scholvin J (2016). Close-packed silicon microelectrodes for scalable spatially oversampled neural recording. IEEE Trans Biomed Eng.

[CR80] Schuettler M, Stiess M, King BV, Suaning GJ (2005). Fabrication of implantable microelectrode arrays by laser cutting of silicone rubber and platinum foil. J Neural Eng.

[CR81] Schuettler M, et al.. Cytotoxicity of platinum black. in: 10th Annual Conference of the International Functional Electrical Stimulation Society. Montreal, Canada. 2005b.

[CR82] Schuettler M. et al.. Interconnection technologies for laser-patterned electrode arrays. Proc IEEE EMBC, Vancouver, Canada. 2008. pp. 3212–15. 10.1109/IEMBS.2008.4649887 19163390

[CR83] Shobe JL, Claar LD, Parhami S, Bakhurin KI, Masmanidis SC (2015). Brain activity mapping at multiple scales with silicon microprobes containing 1,024 electrodes. J Neurophysiol.

[CR84] Sit J, Sarpeshkar R (2007). A low-power blocking-capacitor-free charge-balanced electrode-stimulator chip with less than 6 nA DC error for 1-mA full-scale stimulation. IEEE Trans Biomed Circuits Syst.

[CR85] Sohal H, Vassilevski K, Jackson A, Baker SN, O’Neill A (2016). Design and microfabrication considerations for reliable flexible intracortical implants. Front Mech Eng.

[CR86] Sohal HS (2014). The sinusoidal probe: a new approach to improve electrode longevity. Front Neuroeng.

[CR87] Stieglitz T, Beutel H, Meyer JU (2000). Microflex—a new assembly technique for interconnects. J Intel Mat Syst Str.

[CR88] Suaning GJ, Schuettler M, Ordonez JS, Lovell NH. Fabrication of multi-layer, high-density micro-electrode arrays for neural stimulation and bio-signal recording. in Proc. 3rd Int. IEEE EMBS Conf. on Neural Engineering, Kohala Coast, Hawaii, USA. 2007. pp. 5–8.

[CR89] Takeuchi S, Ziegler D, Yoshida Y, Mabuchi K, Suzuki T (2005). Parylene flexible neural probes integrated with microfluidic channels. Lab Chip.

[CR90] Tand RY (2014). Fabrication of strongly adherent platinum black coatings on microelectrodes array. Sci China Inf Sci.

[CR91] Tyler DJ, Durand DM (1997). A slowly penetrating interfascicular nerve electrode for selective activation of peripheral nerves. IEEE Trans Rehabil Eng.

[CR92] Tyler DJ, Durand DM (2002). Functionally selective peripheral nerve stimulation with a flat interface nerve electrode. IEEE Trans Neural Syst Rehabil Eng.

[CR93] van Dongen MN, Serdijn WA (2016). Does a coupling capacitor enchance the charge balance during neural stimulation? An empirical study. Med Biol Eng Comput.

[CR94] Vanhoestenberghe A, Donaldson N (2013). Corrosion of silicon integrated circuits and lifetime predictions in implantable electronic devices. J Neural Eng.

[CR95] Vetter RJ, Williams JC, Hetke JF, Nunamaker EA, Kipke DR (2004). Chronic neural recording using silicon-substrate microelectrode arrays implanted in cerebral cortex. IEEE Trans Biomed Eng.

[CR96] Vilouras A, Heidari H, Gupta S, Dahiya R (2017). Modeling of CMOS devices and circuits on flexible ultrathin chips. IEEE Trans Electron Devices.

[CR97] Viventi J (2011). Flexible, foldable, actively multiplexed, high-density electrode array for mapping brain activity in vivo. Nat Neurosci.

[CR98] Wang K, Liu CC, Durand DM (2009). Flexible nerve stimulation electrode with iridium oxide sputtered on liquid crystal polymer. IEEE Trans Biomed Eng.

[CR99] Wurth S (2017). Long-term usability and bio-integration of polyimide-based intra-neural stimulating electrodes. Biomaterials.

[CR100] Xie X (2014). Long-term reliability of Al_2_O_3_ and Parylene C bilayer encapsulated Utah electrode array based neural interfaces for chronic implantation. J Neural Eng.

[CR101] Yi W (2015). A flexible and implantable microelectrode arrays using high-temperature grown vertical carbon nanotubes and a biocompatible polymer substrate. Nanotechnology.

[CR102] Zargham M, Glenn Gulak P (2015). Fully integrated on-chip coil in 0.13 μm CMOS for wireless power transfer through biological media. IEEE Trans Biomed Circuits Syst.

[CR103] Zhang T (2009). Flexible electronics: thin silicon die on flexible substrates. IEEE Trans Electron Pa M.

